# Supplementary material for: α-Melanocyte-Stimulating Hormone Attenuates Neovascularization by Inducing Nitric Oxide Deficiency via MC-Rs/PKA/NF-κB Signaling
Source: Int J Mol Sci. 2018 Nov 30;19(12):3823. doi: 10.3390/ijms19123823 (PMC6321109; doi:10.3390/ijms19123823)
Supplement: Supplementary file 1 [file ijms-19-03823-s001.pdf]

Supplementary Figure

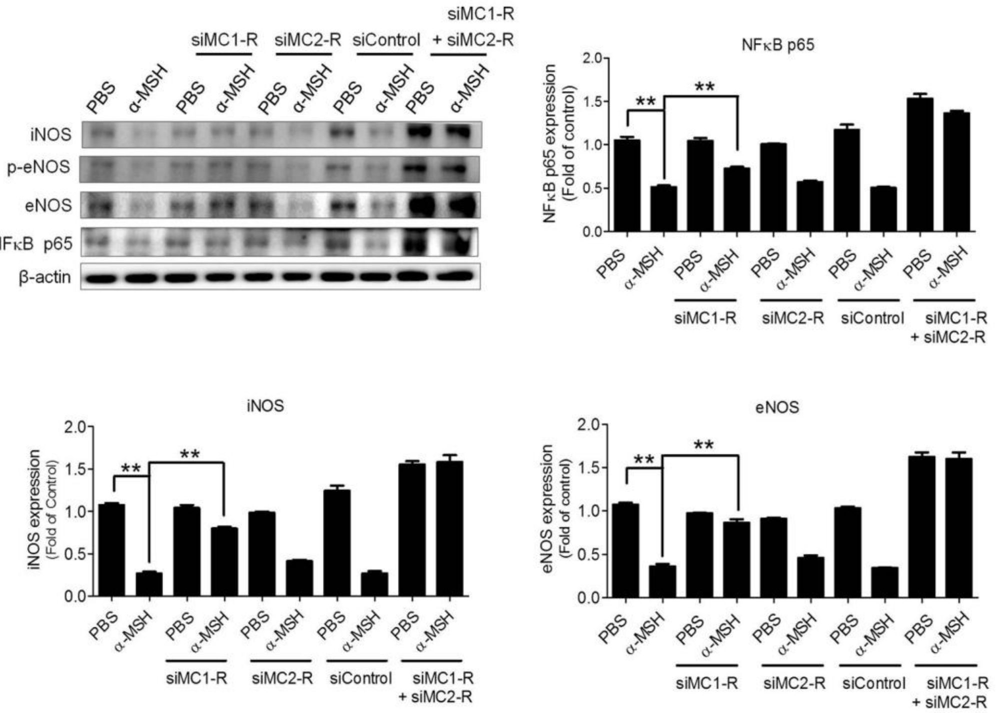

Figure S1. Effect of MC1-R and MC2-R knockdown on iNOS, eNOS and NFκBp65 expression in α-MSH-treated HUVECs

After treatment with α-MSH (10 nM) in the absence or presence of MC1-R and MC2-R siRNA for 24 h, the protein levels of iNOS, p-eNOS, eNOS and NFκBp65 were assessed by immunoblot assay. Data are expressed as mean ± SEM calculated from triplicate experiments which were repeated at least three times. *, $P<0.05$ and **$P < 0.01$.

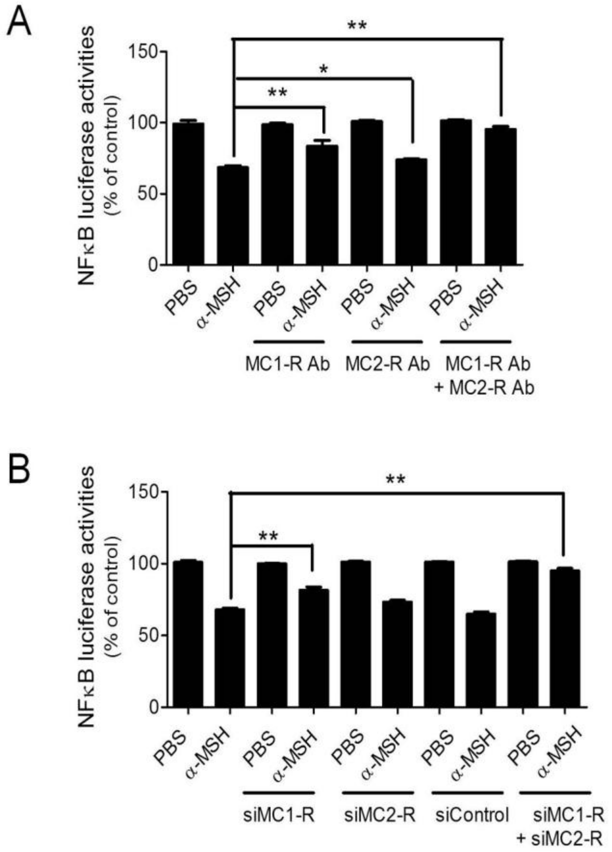

Figure S2. Analyses of α-MSH regulated NFκB signaling in HUVECs

After treatment with α-MSH (10 nM) in the absence or presence of MC1-R and MC2-R (A) antibody and/or (B) siRNA for 24 h, the NFκB promoter luciferase activities reduced by α-MSH can be significantly reversed by blocking MC1-R or MC2-R in HUVECs. Data are expressed as mean ± SEM calculated from triplicate experiments which were repeated at least three times. *, *P*<0.05 and **P < 0.01 compared with the control groups

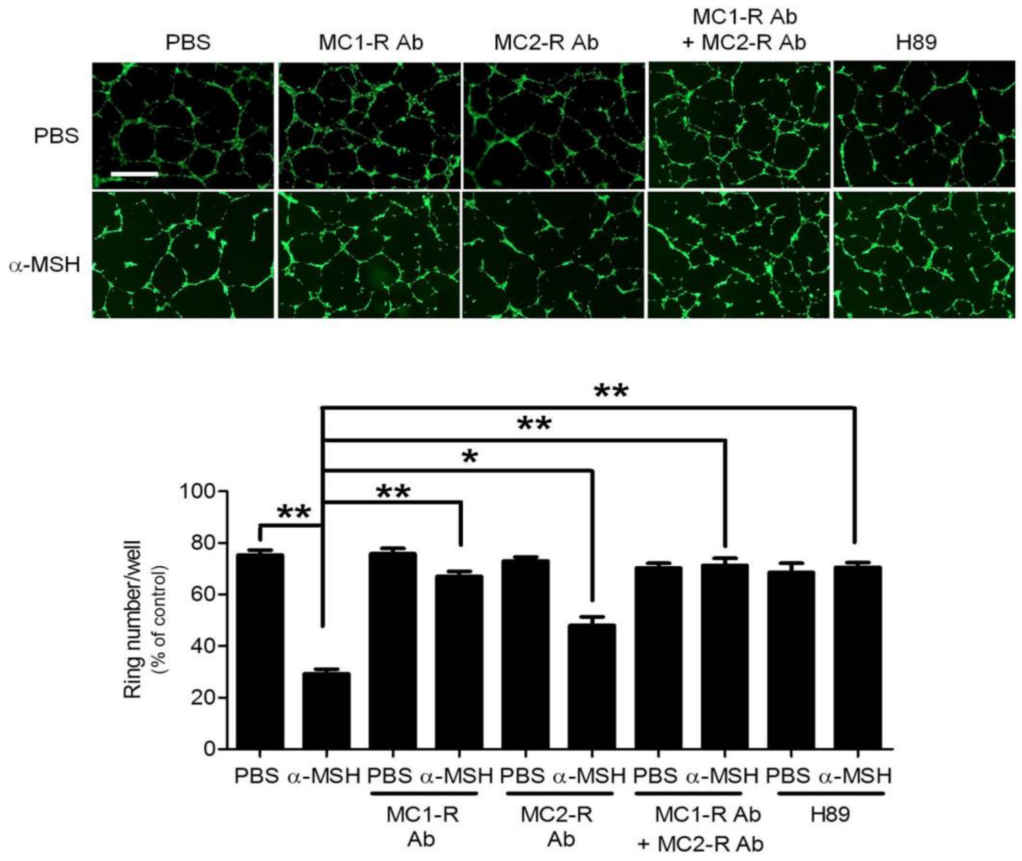

Figure S3. Effects of MC1-R, MC2-R antibodies and H89 on α-MSH–mediated angiogenesis in HUVECs

MC1-R and MC2-R antibody neutralization and H89 treatment attenuated the α-MSH inhibition of tube formation. Data are expressed as mean ± SEM calculated from triplicate experiments which were repeated at least three times. Scale bars, 100 μm.
